# Proton beam therapy for a patient with large rhabdomyosarcoma of the body trunk

**DOI:** 10.1186/s13052-015-0200-0

**Published:** 2015-11-16

**Authors:** Daichi Takizawa, Yoshiko Oshiro, Masashi Mizumoto, Hiroko Fukushima, Takashi Fukushima, Hideyuki Sakurai

**Affiliations:** Departments of Radiation Oncology1 and Child Health3, University of Tsukuba, Ibaraki, Japan; Department of Radiation Oncology, Tsukuba Medical Center Hospital, Ibaraki, Japan; Departments of Child Health, University of Tsukuba and Proton Medical Research Center, Ibaraki, Japan; Proton Medical Research Center, University of Tsukuba, 1-1-1 Tennoudai, Tsukuba, Ibaraki 305-8575 Japan

**Keywords:** Particle therapy, Proton therapy, Pediatric, Rhabdomyosarcoma, Respiratory gating

## Abstract

**Background:**

We present the clinical course of a pediatric patient with large rhabdomyosarcoma of the body trunk who received proton beam therapy (PBT).

**Case presentation:**

A 1-year-old girl was diagnosed with stage IV alveolar rhabdomyosarcoma in 2008. A large tumor was located in the central diaphragm and had infiltrated the liver and pericardium with peritoneal dissemination. Chemotherapy was immediately started with six courses of vincristine, actinomycin-D and cyclophosphamide (VAC) firstly, and secondly followed by 2 courses of ifosfamide, carboplatin and etoposide (ICE), but a large tumor of 15 cm in size remained. The tumor was inoperable because of its location, and photon radiotherapy could not be performed due to limited liver tolerance. The patient was referred to our hospital and received PBT at a dose of 54 GyE in 30 fractions in June 2009.

The tumor quickly responded and 95 % of volume reduction was achieved at the end of PBT. However, marginal recurrence in the caudal part of the irradiated field, where we reduced the proton dose because of the presence of the intestine, was detected in August 2010. The recurrent tumor size was less than 1 cm. Chemotherapy with VAC followed by topotecan and carboplatin (TC) was again tried, but the tumor size was stable. Repeated PBT was not possible because of limited intestinal tolerance; therefore, intraoperative radiotherapy was conducted with 20 Gy of electron beams in April 2011. The tumor was subsequently well controlled, but secondary myelodysplastic syndrome developed and the patient died of hemophagocytic syndrome after umbilical cord blood transplantation in May 2012.

**Conclusion:**

PBT was performed safely and effectively for a 1-year-old girl with alveolar rhabdomyosarcoma with liver and cardiac invasion that was resistant to surgery and chemotherapy. This case illustrates that PBT can be useful in cases that are difficult to treat with conventional radiotherapy.

## Background

Radiotherapy plays an important role in treatment of rhabdomyosarcoma (RMS), with a dose of 40–60 Gy typically required, and hyperfractionation (1.1-Gy twice daily fractions) or standard fractionation (1.8-Gy daily fractions) is used for definitive treatment [1]. The risk of morbidity radiation-related should be carefully considered depending on the volume and the dose delivered in the pediatric patients. We experienced a pediatric patient with an unresectable large alveolar RMS with liver invasion. Photon radiotherapy was considered as the first treatment choice, but was not administered because of the lack of tolerance of the liver. Proton beam therapy (PBT) was chosen in this case, since PBT is a particle radiotherapy with excellent dose localization because of the sharp and narrow Bragg peak [2, 3]. Here, we present the clinical course of the patient after treatment with PBT.

## Case presentation

**Fig. 1:**
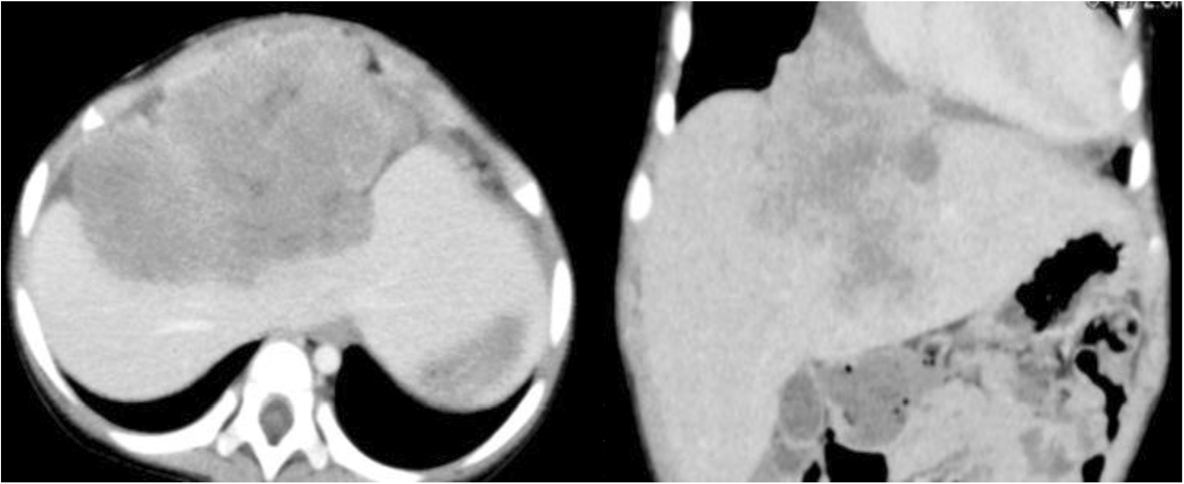
CT images after 6 courses of VAC and 2 courses of ICE, just before the start of PBT

**Fig. 2:**
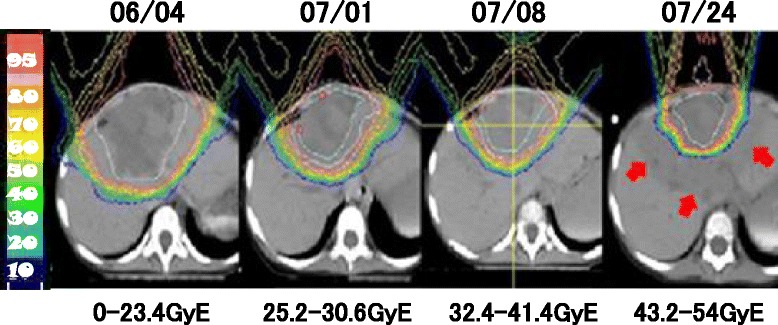
The tumor shrunk during PBT and the treatment field was reduced to fit the tumor size. The white line was clinical target volume. The surrounding low dosing area (arrows) showed acute radiation hepatitis

**Fig. 3:**
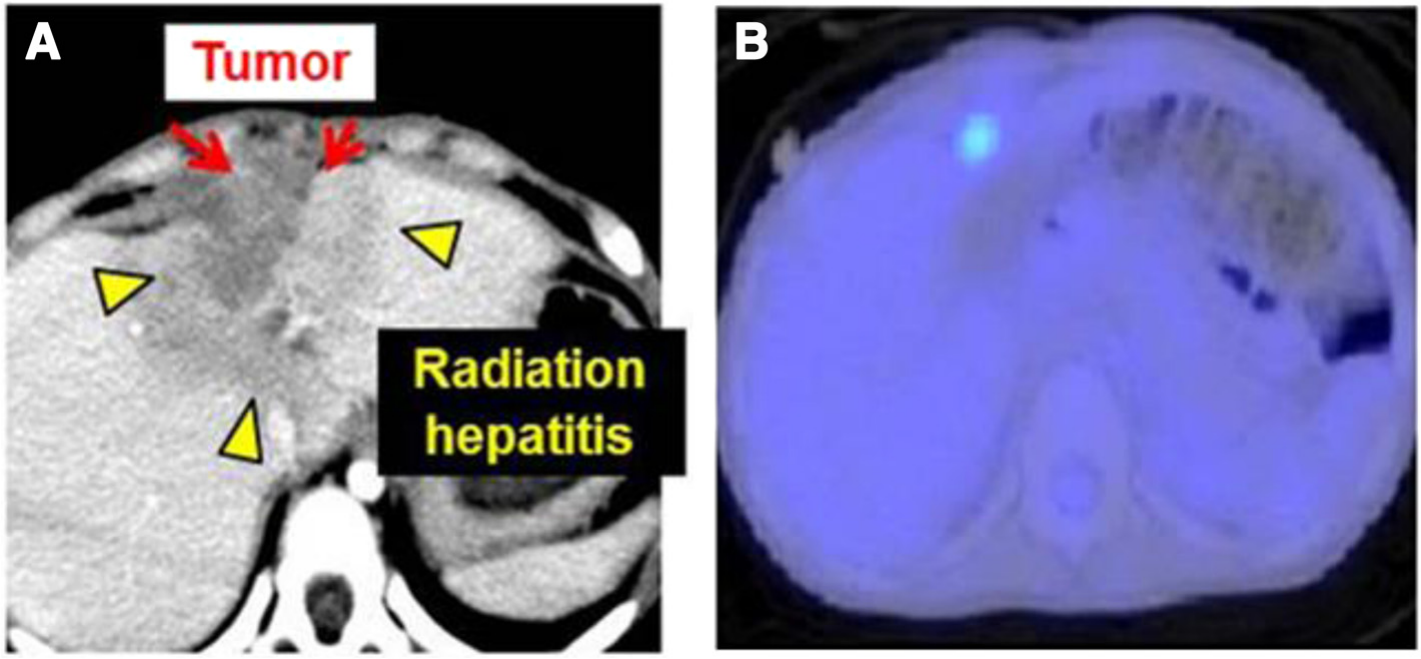
These images were contrast enhanced CT image **a** and PET-CT image **b**, at 1 year after PBT. In figure **a**, local recurrence of the tumor is enhanced at the edge of irradiation, and FDG accumulated same place

**Fig. 4:**
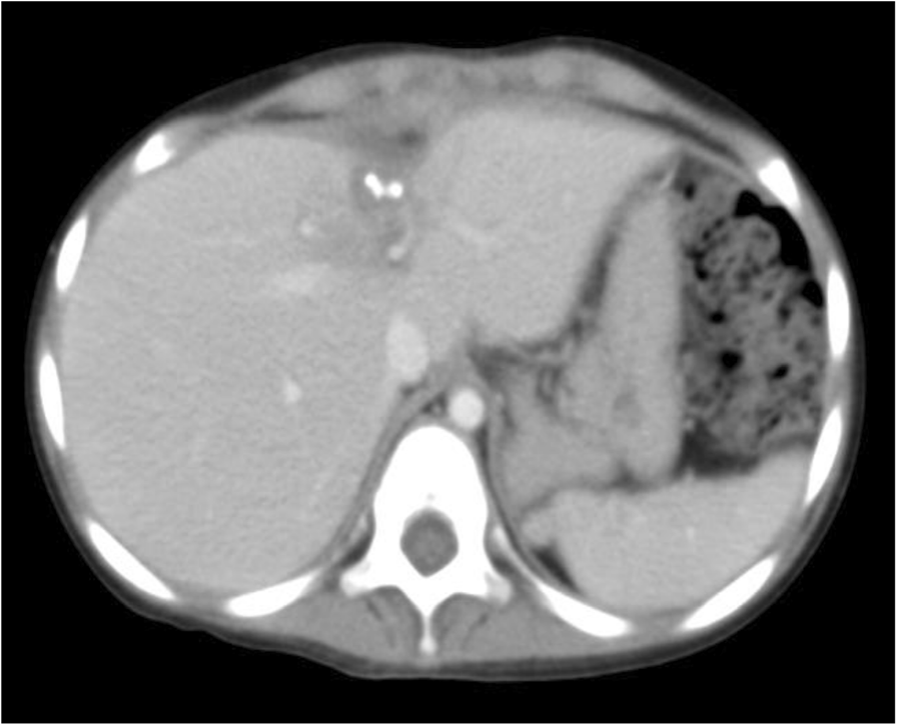
The tumor was well controlled on CT images at 2 months after IORT

A girl aged 1 year and 6 months initially presented with an abdominal mass with a maximum diameter of over 15 cm. The mass was located in the central diaphragm and was infiltrating the liver and pericardium with peritoneal dissemination. Ascites cytology showed class V in Papanicolaou’s classification. The patient was diagnosed as alveolar RMS IRS Stage 4, postoperative group IV, and classified in the high risk group. Chemotherapy was started, but the tumor did not shrink sufficiently: with disease progression after the first 2 courses of VAC every 3 weeks, including vincristine (VCR) + actinomycin-D (Act-D) + cyclophosphamide (CPA); and partial response (PR) after 2 courses of ICE, including ifosfamide (IFM) + carboplatin (CBDCA) + etoposide (VP-16). Surgical resection was not possible for broad tumor’s invasion of the surrounding diaphragm and radical photon radiotherapy could not be performed because the liver could not tolerate the definitive treatment dose. Palliative care was initially recommended from local pediatric oncologists, but subsequently the patient and her family visited our hospital to receive PBT, which was started in June 2009 at age 1 year and 11 months. Peritoneal dissemination disappeared after the chemotherapy, but the tumor was still located in the central diaphragm with infiltration of the liver and pericardium, and the maximum diameter was still over 8 cm at the point (Fig. 1). One month after the last chemotherapy (ICE), a PBT dose of 54 GyE in 30 fractions was applied over a course of 58 days. The relative biological effectiveness (RBE) of the PBT was assumed to be 1.1. We used the passive scattering method for PBT [4]. During PBT, the patient was sedated with anesthesia and immobilized in a body cast. We used a laser displacement sensor (LDS: KEYENCE LB-300) that was the prototype of AZ-733. Respiratory gating in the expiratory phase was used. A respiratory waveform is obtained using a laser range finder that monitors movement of the abdominal surface, and a gating signal is developed. The phase shifts between the respiratory waveform and the 3D tumor motion are principally in the range 0.0 to 0.3 s, regardless of the organ being measured in the system [18]. The gating signal is applied to the accelerator, and the accelerator is triggered within 0.1 s and delivers proton beams. The CTV encompassed the gross tumor volume with a 5- to 10-mm margin in all directions. An additional 5-mm margin was included on the caudal axes to compensate for uncertainty due to respiration-induced hepatic movements. An additional margin of 10 mm was added to cover the entire CTV by enlarging the multileaf collimator and adjusting the range shifter. Proton beams from 155 to 250 MeV generated through a linear accelerator and synchrotron were spread out and shaped with ridge filters, double-scattering sheets, multicollimators, and a custom-made bolus to ensure that the beams conformed to the treatment planning data. The tumor shrunk during PBT, and the treatment field was reduced to fit the tumor size and to keep the intestinal dose within the 50Gy tolerance level [11] (Fig. 2). The tumor showed a good PR (−95 % of the tumor volume) after PBT and the acute toxicity was only Grade 1 radiation hepatitis and dermatitis. After PBT, high dose chemotherapy with VP-16 + CPA + pirarubicine (THP-ADR) + cisplatin (CDDP) + VCR, IFM + VP-16 + Act-D + VCR, and irinotecan (CPT-11) + VCR was continued. The tumor was well controlled for 1 year with regular follow-up MR or CT scans performed every 3 months, but then recurred at the edge of the irradiation field where the irradiation dose was reduced due to the proximity to the intestine (Fig. 3). The chemotherapy regimen was changed to low dose VAC, but the tumor volume remained stable. Repeated radiotherapy was considered, but could not be administered due to intestinal tolerance. Therefore, tumor excision with intraoperative radiotherapy (IORT) using an electron beam of 20 Gy was conducted in May 2011, at age 3 years and 10 months. After IORT, the tumor was well controlled and a CT image showed only radiation hepatitis without a tumor (Fig. 4). However, the patient developed secondary myelodysplastic syndrome (MDS) in December 2011, and died of hemophagocytic syndrome after umbilical cord blood transplantation in May 2012.

## Discussion

**Fig. 5:**
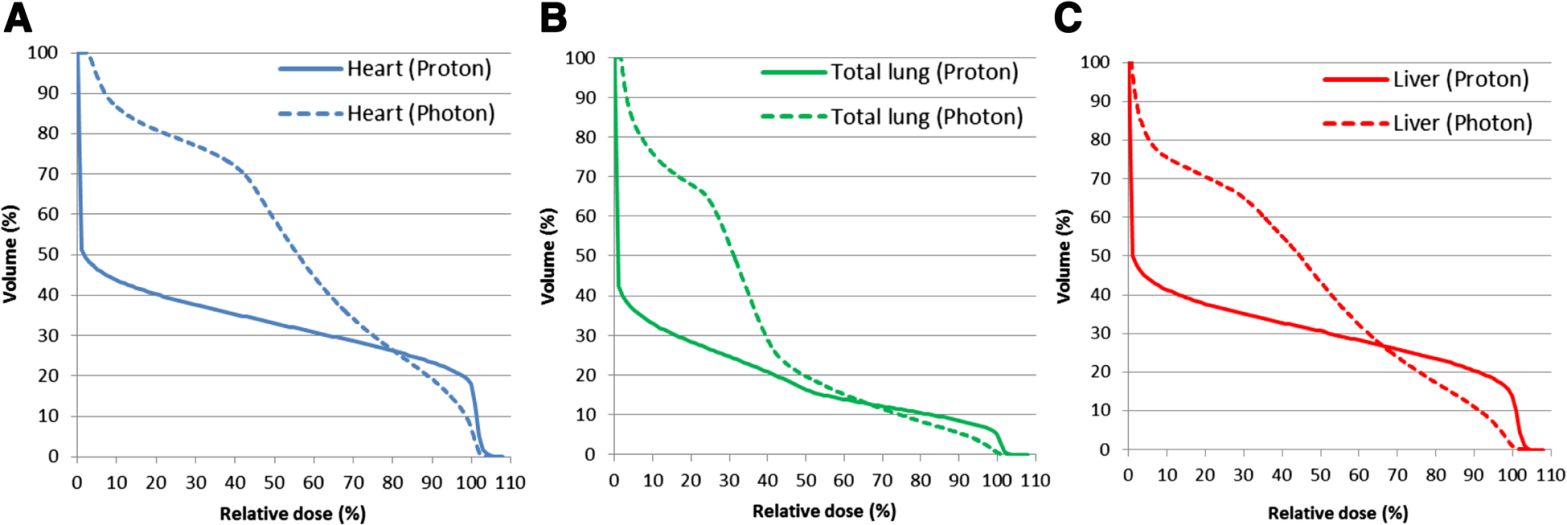
These graphs showed DVHs of PBT and IMRT for OARs of heart (**a**), total-lung (**b**), and liver (**c**). PBT reduced low-dose area compared with IMRT

The incidence of soft tissue sarcomas in children and adolescents younger than 20 years of age is 11.0 per million, representing 7.4 % of cancer cases for this age group [5]. Rhabdomyosarcoma is the most common soft tissue sarcoma in children aged 0–14 years, representing nearly 50 % of soft tissue sarcomas for this age range, with an incidence rate of 4.6 per million. There are two major types of rhabdomyosarcoma: the embryonal one which occur in 75 % of the cases and alveolar [6]. The incidence of embryonal rhabdomyosarcoma is higher among children aged 0–4 years, while the alveolar subtype incidence is similar throughout childhood [5]. The embryonal type has a better prognosis than the alveolar type [7]. The standard therapy for pediatric rhabdomyosarcoma is multiagent chemotherapy and local therapy of surgery with or without radiotherapy. Only 15 % of patients with completely resected embryonal rhabdomyosarcoma have a good outcome without radiotherapy, and thus radiotherapy is used in most cases. The radiotherapy dose mainly depends on the amount of residual disease, if any, after primary surgical resection, with doses increased from 36 to 50.4 Gy as the risk increases [8–10]. In our case, the tumor widely involved the liver, and this prevented definitive radiotherapy because of the low tolerance of radiation doses of 30, 35, and 50 Gy for the total, two-thirds, and one-third of the liver, respectively [11]. For comparison, we planned intensity-modulated radiation therapy (IMRT) using the same conditions as those used for PBT. In the IMRT plan, the liver volume irradiated with ≥20 Gy (V20) was 60 % at a treatment dose of 54 Gy, compared to 34 % in PBT. PBT reduced low dose area and was expected [Fig. 5]. The total liver volume was 500 cc and the remnant volume was only 200 cc, which made it difficult to administer photon radiotherapy in the present case. Surgery was also not possible for broad tumor’s invasion of the surrounding diaphragm and chemotherapy alone was ineffective. Palliative irradiation was also recommended, but survival was estimated to be less than one year with palliative therapy. Proton beams have a Bragg peak in which the dose rapidly falls off at the end of the beam range at a depth within the patient. For this reason PBT can deliver a high dose to the limited volume of a liver tumor while non-cancerous liver tissue is only exposed to very low doses. PBT also allows preservation of a larger volume of normal liver tissue compared to photon radiotherapy. Based on these advantages, we have treated many patients with liver malignancies [12–14]. Therefore, we thought that PBT was reasonable and might be effective in the present case, even though we had not previously used this approach for pediatric RMS widely located in the liver. Several reports have described the advantages of PBT compared to photon radiotherapy, including IMRT. Cotter et al. found that proton beams can reduce the dose to normal organs adjacent to the tumor, such as the bladder, testis and bones, compared to IMRT in radiotherapy for bladder or prostate rhabdomyosarcoma [15]. In spot-scan PBT for pediatric malignant soft tissue tumor including rhabdomyosarcoma, Timmerman et al. found high tolerability of PBT and suggested that IMRT requires a wider field compared to PBT; therefore, PBT results in lower doses to the volume around the target and the secondary cancer risk is decreased [16]. In a phase II study comparing PBT and IMRT, Ladra et al. reported favorable disease control and dose distribution [17]. The tumor recurrence 1 year after PBT in our case may have been due to field shrinkage to reduce the dose to the gastrointestinal tract after 41.4 GyE. In adults, a dose of about 50 GyE may be acceptable, but radiation sensitivity is higher in pediatric patients and may have been further enhanced by concurrent chemotherapy in the present case. For these reasons, we reduced the small bowel dose after administration of 41.4 GyE. The recurrence was well controlled by IORT. Unfortunately, the patient died of a regimen-related toxicity of unrelated cord blood transplantation during treatment for secondary MDS. However, PBT seemed to be effective with a good tolerance to the radiation treatment and with few acute side effects (G1 hepatitis), and potentially beneficial with IORT to improve the local control. In conclusion, we suggest that PBT could be a valide and safe alternative technique to be considered for pediatric patients with RMS who cannot receive definitive photon radiotherapy.

## Conclusion

In this case, PBT was performed safely and effectively with alveolar rhabdomyosarcoma with liver and cardiac invasion that was unable to be removed with surgery and was resistant to chemotherapy. We supposed that side effect and tumor’s local control turned worse, if we used conventional radiotherapy not PBT. This case illustrates that PBT can be useful in cases that are difficult to treat with conventional radiotherapy.

## Consent

Written informed consent was obtained from the patient’s legal guardians for publication of this case report and any accompanying images. A copy of the written consent is available for review by the Editor-in-Chief of this journal.
